# Building inner ears: recent advances and future challenges for in vitro organoid systems

**DOI:** 10.1038/s41418-020-00678-8

**Published:** 2020-12-14

**Authors:** Wouter H. van der Valk, Matthew R. Steinhart, Jingyuan Zhang, Karl R. Koehler

**Affiliations:** 1 Department of Otorhinolaryngology and Head & Neck Surgery, Leiden University Medical Center, Leiden, Netherlands; 2 Department of Otolaryngology, Boston Children’s Hospital, Boston, MA 02115 USA; 3 Department of Otolaryngology-Head and Neck Surgery, Indiana University School of Medicine, Indianapolis, IN 46202 USA; 4 Medical Neuroscience Graduate Program, Indiana University School of Medicine, Indianapolis, IN 46202 USA; 5 Department of Plastic and Oral Surgery, Boston Children’s Hospital, Boston, MA 02115 USA; 6 F.M. Kirby Neurobiology Center, Boston Children’s Hospital, Boston, MA 02115 USA; 7 Department of Otolaryngology-Head and Neck Surgery, Harvard Medical School, Boston, MA 02115 USA

**Keywords:** Cell biology, Somatic system, Stem-cell research

## Abstract

While inner ear disorders are common, our ability to intervene and recover their sensory function is limited. In vitro models of the inner ear, like the organoid system, could aid in identifying new regenerative drugs and gene therapies. Here, we provide a perspective on the status of in vitro inner ear models and guidance on how to improve their applicability in translational research. We highlight the generation of inner ear cell types from pluripotent stem cells as a particularly promising focus of research. Several exciting recent studies have shown how the developmental signaling cues of embryonic and fetal development can be mimicked to differentiate stem cells into “inner ear organoids” containing otic progenitor cells, hair cells, and neurons. However, current differentiation protocols and our knowledge of embryonic and fetal inner ear development in general, have a bias toward the sensory epithelia of the inner ear. We propose that a more holistic view is needed to better model the inner ear in vitro. Moving forward, attention should be made to the broader diversity of neuroglial and mesenchymal cell types of the inner ear, and how they interact in space or time during development. With improved control of epithelial, neuroglial, and mesenchymal cell fate specification, inner ear organoids would have the ability to truly recapitulate neurosensory function and dysfunction. We conclude by discussing how single-cell atlases of the developing inner ear and technical innovations will be critical tools to advance inner ear organoid platforms for future pre-clinical applications.

## Introduction

Over 6% of people worldwide suffer from hearing loss [1] and likewise 6% suffer from balance disorders [2]. Both these sensory systems are located in the inner ear, which can be affected by the aging process, genetic mutations, infectious diseases, chronic infections, noise exposure, and ototoxic drugs [1, 2]. Despite the prevalence of inner ear sensory dysfunction, which in the case of hearing loss is irreversible, there are currently no approved medications specifically targeting sensory recovery. Devices, such as hearing aids or cochlear implants, are commonly used to manage—not cure—moderate to severe hearing loss cases. In the hunt for new therapies, in vitro human, three-dimensional and multicellular systems mimicking the inner ear, that is inner ear organoids, could be a useful tool to accelerate therapeutic discovery. Such models could be used to test virus-mediated gene therapies for congenital hearing loss disorders [3, 4], and screen for compounds to regenerate sensory cells following ototoxic degeneration [5]. Additionally, human organoid models will provide insight into unique human aspects of inner ear development and pathologies, which are impossible to gain from animal studies. Despite several recent breakthroughs, application of cellular models of the inner ear have been slow to gain momentum for disease models and as drug discovery platforms. In this perspective article we will briefly discuss progress toward in vitro inner ear modeling approaches, which has been thoroughly covered in recent reviews [3, 6–11]. Then, we will focus on diagnosing key barriers to progress and discuss advances from other fields that shed light on how more complete, scalable, and reproducible inner ear organoid models can be built.

## The challenge of modeling inner ear development in vitro

Different approaches of modeling the human inner ear in vitro have been demonstrated using human pluripotent stem cells (hPSCs) [12–24], adult tissue resident stem cells [25], or fetal progenitor stem cells [26, 27]. Unlike some other organ systems (e.g., the intestines), the routine use of patient-derived tissue for research is not scalable, because the inner ear is difficult to biopsy and refractory to long-term culture [28, 29]. Furthermore, the use of fetal tissue is tightly regulated in some countries and the unpredictability of specimen collection complicates downstream analysis [27]. In contrast, hPSCs are potentially an endless source of cellular tissue for experimentation and can be genetically manipulated (e.g., introduction of pathologic genetic mutations, or the generation of reporter cell lines). There are two types of hPSCs: embryonic stem cells (ESCs) derived from pre-implantation embryos and induced pluripotent stem cells (iPSCs) derived from reprogrammed adult cells. hPSCs can be differentiated to otic progenitor cells and more mature inner ear cell types by mimicking embryonic and fetal development. These differentiation approaches have been recently reviewed [7, 8, 10]. In the embryo, inner ear development requires the assembly of diverse cell types from multiple cell lineages: the epithelial, neuronal, and glial cells of the inner ear are derived from the ectoderm germ layer, whereas the specialized periotic mesenchyme (POM) that surrounds the inner ear arises from the mesoderm germ layer and cranial neural crest—a population of cells that generate neurons, glia, melanocytes, and mesenchyme in the head and neck [30]. A major long-term bioengineering challenge is to incorporate all of these multiple cell lineages into an inner ear organoid in vitro.

**Fig. 1:**
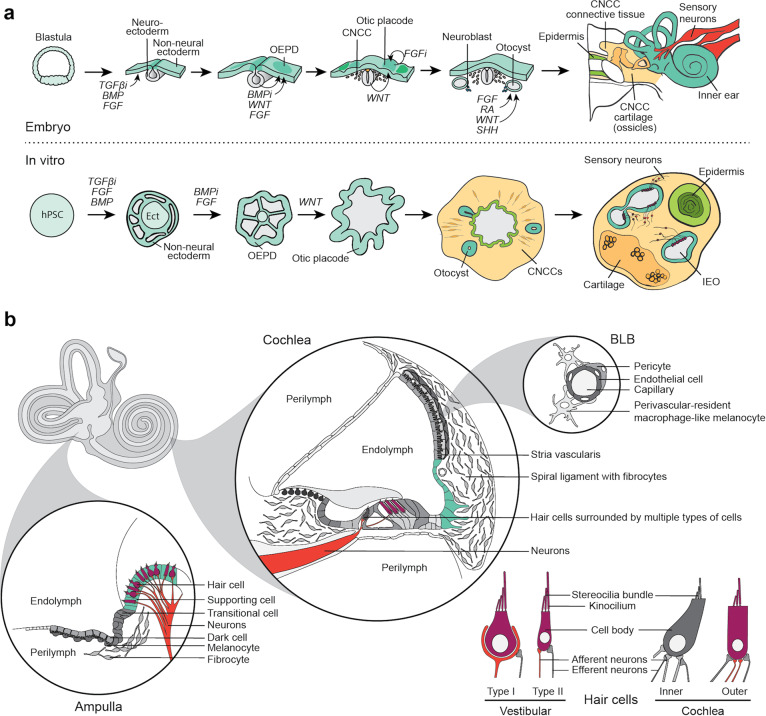
Schematic overview of embryonic inner ear development and inner ear cell types generated from hPSCs in vitro. **a** Embryonic development of the inner ear (top) is mimicked in vitro to generate IEOs from PSCs (bottom) [21]. Top: during gastrulation of the blastula, neuroectoderm and non-neural ectoderm arises. Decreased TFGß expression and increased BMP and FGF signaling stimulate non-neural ectoderm formation. Within the non-neural ectoderm, the OEPD is formed due to diminished BMP expression, in addition to elevated FGF and WNT signaling levels. Continuing WNT activation as well as decreased FGF signaling gives rise to the otic placode within the OEPD. After invagination and formation of the otocyst, further patterning occurs by a combination of FGF, RA, WNT, and SHH signaling modulations, eventually giving rise to the inner ear. Bottom: PSCs are differentiated in a similar way, in which TFGß inhibition, together with FGF and BMP signaling activation, give rise to a non-neural ectoderm on the surface of the hPSC aggregate. With BMP inhibition and FGF signaling, the OEPD is formed in this surface ectoderm. Subsequent stimulation of WNT signaling gives rise to the otic placode within the OEPD. The placode invaginates to form otocysts that subsequently mature to IEOs. Other types of tissue, including cartilage and skin, are also induced within the aggregate. CNCC: cranial neural crest cells. **b** Schematic of the vestibular and cochlear cell types. Cell types described to be generated from hPSCs are colored. Ampulla: cross-section of one of the three ampullae of the semicircular canals as an example of the vestibular system. Vestibular hair cells in purple [21, 22, 24], supporting cells in aqua [15, 21–24], and neurons in red [12–14, 16, 19, 21–24] are described to be generated from hPSCs. The non-sensory cell types (in gray) are not described. Cochlea: a cross-section of the basal turn of the cochlea showing multiple cell types, with only neurons [12–14, 16, 19, 21–24], outer hair cells [22] and outer sulcus cells [20] reportedly generated from hPSCs. BLB: blood-labyrinth barrier, the barriers between the vasculature and inner ear fluids are present in both the vestibular system and cochlea, which have not been generated yet. Hair cells: Both type I [22] and type II [21, 24] vestibular hair cells, as well as outer cochlear hair cells are described [22]. Synaptic processes are also depicted [12, 13, 19, 21, 22].

During early embryogenesis, pluripotent cells in the epiblast generate the ectoderm germ layer, which splits into the non-neural (also called surface) ectoderm and neuroectoderm. The otic placode arises at the border region between the cranial non-neural ectoderm and neuroectoderm, known as the otic-epibranchial placode domain (OEPD) [31, 32]. Several signaling pathways, including fibroblast growth factors (FGFs), WNT, transforming growth factor-beta (TGF), bone morphogenetic proteins (BMPs), sonic hedgehog (SHH), and retinoic acid (RA), are involved in this early otic cell fate specification. TGF, FGF, and WNT signaling appear to be the most essential morphogenic cues to form the OEPD in the embryo [33–35]. During otic placode formation, WNTs secreted from cranial mesenchyme and neural tube activate NOTCH pathway signaling in the surface ectoderm [36]. A negative feedback loop in turn, downregulates FGF signaling to further specify otic progenitors within the placode [37]. The otic placode subsequently invaginates to form the otocyst (also known as the otic vesicle), which gives rise to the majority of epithelial cell types in the vestibular and cochlear compartments of the inner ear. In addition to otic placode derived-cells, lineage tracing experiments in chickens and mice have shown that a limited number of neural crest cells contribute to the otocyst epithelium; however, it remains unclear what contribution these cells have to non-sensory and sensory epithelia in the inner ear later in development [38, 39]. Based on these developmental biology studies, a consensus model of otic induction from PSCs has emerged in recent years (Fig. 1a). Induction of otic and other cranial placodes, however, show limited efficiency in comparison to other ectodermal lineages in two-dimensional monolayer cultures [40–42]. In our previous study, we have shown that multiple otic placodes and otocyst-like structures can be generated in vitro from a three-dimensional (3D) hPSC aggregate by modulating TGF, BMP, FGF, and WNT signaling and extracellular matrix-related mechanical interactions (Fig. 1a) [21]. The floating 3D culture appears to be ideal for allowing differentiating ectoderm and otic lineage cells to undergo the complex morphogenetic changes required for placode generation. Interestingly, using a modified version of our otocyst culture system, we recently demonstrated the generation of hair-bearing skin organoids—like the inner ear, hair follicle induction involves formation of epithelial placodes [43]. Our understanding of the biomechanics of epithelial placode induction is limited and worthy of future exploration to gain insight into how to improve in vitro placode induction approaches [44–46].

Upon embryonic otocyst formation, asymmetric gene expression leads to dorsoventral and anteroposterior patterning that sets up the coordinates for subsequent development of the semicircular canals, endolymphatic sac, vestibular organs, and cochlea [47]. Signals from surrounding tissues—the notochord, neural tube, and cranial mesenchyme—are crucial for setting up the axes of the otocyst [48]. SHH secreted from the notochord and neural tube floor plate has been shown to establish the ventral part of the otocyst, giving rise to the cochlea, saccule and vestibulocochlear ganglion [49–51]. The graded dorsoventral SHH signaling is accomplished by a competing mechanism between GLI activators and GLI repressors. SHH knockout experiments have shown that attenuation of SHH within the mouse otocyst has little effect on the development of dorsal tissues, such as the semicircular canals, ampullae and utricle, but in contrast has a detrimental effect on ventral induction, highlighting the importance of SHH signaling for cochlea formation [52]. Meanwhile, the WNT and BMP signaling molecules have been shown to act as dorsalizing cues in the mouse otocyst [53]. The neurosensory region of the otocyst gives rise to delaminating neuroblasts and the sensory epithelia. Formation of this region is mediated by NOTCH and SOX2 activity downstream of WNT signaling [54]. Division of the neurogenic and prosensory zones of the otocyst is mediated by transcription regulators, such as TBX1––a negative regulator of neurogenesis that acts downstream of SHH, WNT, and RA signals [55]. Following otocyst patterning, the anteroventral prosensory domain begins to elongate to form the cochlear duct, and eventually forms the basal portion of the organ of Corti in the cochlea. During elongation and coiling of the cochlear duct, elaborate signaling dynamics fine tune the cellular diversity of the organ of Corti. The boundary between organ of Corti’s Kölliker organ (neural) and outer sulcus is regulated by levels of BMP4 expression; cells exposed to moderate levels of BMP4 signaling will adopt a prosensory cell fate, whereas under higher levels, cells will adopt a non-sensory cell fate [56]. Extrinsic SHH signaling arises from the cochleovestibular ganglion, and the inhibition of this signal stimulates cochlear prosensory expansion [57]. In addition, FGF signaling is essential to regulate the width of the prosensory domain [58], and NOTCH plays a role in lateral induction of cochlear sensory cells [59, 60]. A recent study has also revealed a role for Hippo signaling in progenitor cell renewal, thus controlling the size of the organ of Corti [61]. Similarly, it has been shown that patterning of the vestibular system occurs by coordinated interactions of  the NOTCH, WNT, BMP, FGF, and RA signaling pathways [62–66]. Strikingly little is known about the signaling mechanisms that underlie non-sensory epithelial specification in the ear; however, retinoic acid seems to be involved in the formation of non-sensory ionic regulatory epithelia in both the cochlea and vestibular end organs [67, 68].

Thus, the inner ear is patterned through the integration of a multitude of signaling pathways across space and time. These signals arise from within epithelia and from surrounding tissues, which enables the differentiation of otic progenitor cells to subsequent cochlear and vestibular fates. Our collective knowledge of these mechanisms has come from animal models and few, if any, mechanistic studies have been performed on human fetal inner ear tissues (see Roccio et al. [27] for an exception). To some degree, spatiotemporal signaling cues can be mimicked in hPSC three-dimensional cultures using bath application of recombinant proteins and small molecules, which can stimulate self-assembly of inner ear epithelia and neuron complexes (as demonstrated in Koehler et al. [21]); however, this approach is difficult to control and the generated organoids take on irregular shapes/sizes and contain an unpredictable mix of sensory and non-sensory cells. In future studies, more sophisticated 3D bioprinting and/or microfluidic-based approaches may be necessary to establish spatially controlled cellular structures that can be acted on by signaling gradients—resulting in an inner ear organoid-on-chip. Recent studies using microfluidic or microwell systems to pattern hPSCs into multilineage embryo-like, renal, or intestinal structures could be a guide toward improving control and reproducibility of otic induction [69–72].

## Constructing inner ear organoids: what cells are missing?

The cellular composition of the inner ear is remarkably diverse, containing over fifty distinct cellular subtypes, including hair cells, supporting cells, non-sensory epithelial cells, as well as unique neurons and mesenchymal populations [73, 74]. Current hPSC-derived models only appear to contain a small subset of these cell types or, perhaps, enclose greater cellular diversity that has not been sufficiently characterized [12–24] (Fig. 1b). Due to their prevalence in human inner ear disorders, the main focus of characterization has been on neurosensory cell types. For instance, the hair cells generated from hPSCs to date are predominantly vestibular in character [13, 15–17, 21–24]. In correspondence with the neurosensory bias in defining these models, neurons [12–14, 16, 19, 21–24] and glial cells are described [12, 19, 21], with some studies describing synapse formation between the generated hair cells and neurons [12, 13, 19, 21, 22]. However, a clear distinction between vestibular and cochlear neurons has not been made; thus, we do not yet have a complete picture of native inner ear cell types that are recapitulated in inner ear models.

We contend that fully defining the cell populations of inner ear organoid models will be important for properly mimicking human inner ear disorders in vitro. Although a large subset of the >150 genes associated with hereditary deafness impact the function of hair cells, many of these genes are expressed in non-sensory or mesenchymal cell populations [75]. Recent studies using mPSCs prove the capability of studying hearing loss caused by dysfunction of Gap Junction Protein Beta 2 (GJB2) [76], BarH Like Homeobox 1 [77], and Transmembrane Protease Serine 3 (TMPRSS3) [78]. Moreover, hiPSCs have been used to study MYO15A [79] and MYO7A [80], as well as SLC26A4 [20, 81]. These studies, however, focus on specific neurosensory-related cell types, including hair cells [77, 79, 80], cochlear supporting cells [76], and cochlear outer sulcus cells [20], rather than describing a functional unit containing multiple inner ear cell types. Overall, the neurosensory bias of the PSC-related literature makes it unclear to what extent PSC-derived inner ear cells or organoids reflect the true cellular diversity of the inner ear. Thus, the potential disease modeling applications are limited. For instance, there are cochlear and vestibular diseases, as well as certain ototoxic agents, that have their pathological impact—either directly or indirectly—on non-sensory cells of the inner ear [4, 75, 82–85]. Therefore, it will be essential to generate inner ear models that contain a diverse functional unit of inner ear cell types to capture complex genetic or drug-related mechanisms. In the following sections, we will provide more insight into the different cell types forming the functional unit of the inner ear and give directions for future organoid research.

### Epithelia

A primary goal of inner ear organoid generation is the production of hair cells. Although the majority of hair cells are reported to be vestibular-like [21, 22, 24], one report suggests the presence of cochlear-like outer hair cells based on the expression of SLC26A5 [22]. Further investigation into the specific subtype of these presumptive cochlear outer hair cells must be established by functional analysis or reviewing additional protein expression, bearing in mind the temporal and spatial expression of certain hair cell markers [86]. Additionally, hair cells do not function on their own; normal hair cell function is dependent on the endolymphatic electrical potential, which is maintained by the surrounding ionic regulatory epithelia [87], a subset of non-sensory epithelial cells. These epithelia include the stria vascularis in the cochlea and the dark cell area in the vestibular organs, which function together with surrounding epithelia and POM in ion recycling and endolymph homeostasis [87]. Cell types within these ionic regulatory epithelia have distinct embryonic origins, which will complicate our ability to model them in vitro. In the stria vascularis, marginal cells arise from the otocyst, intermediate cells from the neural crest, and basal cells from the POM [88–90]. Similarly, the vestibular dark cells arise from the otocyst and associated melanocytes from the neural crest [91]. Dysfunction of these epithelia can lead to metabolic hearing loss and vestibular dysfunction [92, 93]. The ionic regulatory cell types have not been described yet in hPSC-derived inner ear models. Our group recently showed that melanocytes arise in a skin organoid model that shares many induction steps and cellular components with the inner ear organoid model [43]; thus, it may be possible to co-induce the otocyst-derived and neural crest-derived components of ionic regulatory epithelia.

Cochlear outer sulcus-like cells, as part of the non-sensory epithelia, could be derived from human iPSCs [20]. The sulcus-like cells were identified by their expression of various markers, including the anion exchanger SLC26A4. However, these markers are expressed in a variety of epithelial cells throughout the inner ear, such as the endolymphatic sac, the vestibular transitional cells, and outer-sulcus cells [94]. Moreover, cell type-specific proteins might be expressed by different cell types during fetal development before becoming restricted to a specific cell type at a later developmental stage [89, 91, 95]. In future work, it will be beneficial to find markers that differentiate between these cell populations before assigning a definitive cochlear, vestibular, or endolymphatic identity. In general, the field should make use of techniques, such as single-cell RNA sequencing or mass cytometry, instead of relying on single marker genes or proteins to define inner ear cell fates that undergo dynamic spatiotemporal changes during development.

### Neurons and glia

The formation and maintenance of synapses between hair cells and spiral ganglion neurons (the cochlear neurons) plays an important role in functional development and is linked with noise-induced hearing loss [96]. Recent studies have demonstrated that inner ear spiral ganglion-like neurons can be generated from hPSCs in 2D culture [19] as well as the organoid model [21]. However, it remains unclear how closely neurons in these systems mimic the gene expression and functional signatures of bone fide spiral ganglion neurons. The inner ear arises in a milieu rich with sensory neurons (cranial nerves V, VII, VIII, IX, and X) derived from placodes (the otic, epibranchial, and trigeminal placodes) and cranial neural crest cells; thus, it is critical to distinguish between these other possible neuron subtypes during the PSC derivation process. To date, a lack of reference data and limited markers has made it difficult to discern neurons of otic, epibranchial, and neural crest origin. Recent single-cell RNA-sequencing studies on various head and neck peripheral neurons should be a valuable resource to better define PSC-derived otic neurons relative to other cranial neurons [97–99]. Beyond characterizing molecular signatures, it will be important to elucidate the function properties of organoid neurons. It is well known that spontaneous activity occurs in the auditory and vestibular organs during development [100]. Recently, independent research groups linked spontaneous activity in hair cells to cell fate specification in nascent spiral ganglion neurons [101, 102]. It will be insightful to confirm that spontaneous activity is present during organoid maturation and also to validate that organoid-derived neurons undergo subtype specification in a similar manner to the native inner ear.

Schwann cells are the myelinating cells of peripheral nerves, including the VIII cranial nerve in the inner ear. Unlike inner ear neurons, which arise from the otocyst, Schwann cells arise from the cranial neural crest. Remarkably, the inner ear organoid model appears to co-produce S100B + Schwann cells that closely associate with sensory neurons in the culture (Fig. 1a) [21]. The characterization of Schwann cells in this model remains limited. For instance, it is not known whether organoid Schwann cells actually begin to express myelin basic protein (MBP) and myelinate organoid neurons. It will be important to further elucidate the identity and function of Schwann cells in the inner ear organoid system.

Organoids could also be used to investigate neural pathfinding and synapse formation of otic afferent neurons with central nervous system targets. A co-culture system of a developing inner ear organoid paired with developing hindbrain tissue, would potentially mimic elements of the otic ganglion-to-cochlear nucleus circuit [103]. The type of complex interactions between the developing inner ear and hindbrain could be captured in this system in a similar manner to that performed with dorsal and ventral cerebral organoids in recent studies [104]. A key barrier to building an inner ear-hindbrain organoid system is our lack of knowledge about the mechanisms needed for brainstem and, specifically, cochlear nucleus induction from pluripotent stem cells. Efforts toward single-cell mapping of neurons in the brainstem nuclei over developmental time, like a recent study on dorsal raphe neurons, will be critical for future progress [105].

### Periotic mesenchyme (POM)

In addition to the role of POM in endolymph ionic homeostasis, it plays an important role in otocyst patterning [48]. Shortly after invagination of the otic placode, at around E9.5 in mice or fetal week 4–5 in humans, the POM begins to form at the anteroventral pole of the otocyst [106]. The POM is a specialized type of cranial paraxial mesoderm and neural crest [30], which expresses specific transcription factors, TBX1, TBX18, and POU3F4 [106–109]. Many of these genes are associated with deafness and/or vestibular dysfunction [67, 108, 110]. Over five different types of fibrocytes with unique protein expression and spatial localization arise from the POM [92, 93]. The POM gives rise to the cartilaginous and, later, bony otic capsule, as well as the temporal bone. Clearly, the POM is a critical component of normal inner ear morphogenesis. A detailed understanding of what types of mesenchymal cells arise in inner ear organoid cultures is lacking. Our published data suggest that inner ear organoid epithelia co-develop with a neural crest-derived mesenchyme that produces fibrocyte-like cells and cartilage, similar to the POM [21] (Fig. 1a). However, it is not known whether authentic POM—expressing the transcription factors mentioned above—arise in these cultures or whether the mesoderm-derived components of the POM are present. It will be important to define the organoid mesenchyme for future progress. Notably, the importance of organ-specific mesenchyme for higher-order organoid development and maturation has been highlighted in several recent publications on kidney, gut, and skin organoids [43, 111–113]. In particular, the organoid model could be used to study how reciprocal interactions between POM and epithelia impact specification and maturation of cochlear versus vestibular cell fates and cell type specification.

### Vasculature and immune cells

The inner ear has a unique relationship to the rest of the body. In many ways it is isolated: largely immune privileged and with a vascular barrier system called the blood-labyrinth barrier (akin to the blood-brain barrier (BBB) in the central nervous system) [114]. It is unclear what role the vasculature plays in inner ear organogenesis; however, blood flow through capillaries in the stria vascularis appears to be integral to maintenance of the endocochlear potential [115]. An often-cited weakness of organoid models is the lack of vasculature; however, recent work has led to blood vessel organoids and incorporation of a BBB in cerebral organoids [116–118]. Likewise, these novel platforms could be leveraged to infuse inner ear organoids with endothelial cells and pericytes to investigate the role of vasculature in inner ear organ maturation. Incorporation of vasculature seems to enhance maturation of intestinal and kidney organoid systems [119–121]. Homan et al. shows that the introduction of endothelial cells and media flow actually enhances maturation in kidney organoids compared to static controls; however, the presence of vasculature alone was not enough to achieve maturation [119]. Additionally, Palikuqi et al. recently described an organoid-compatible system with perfusable vessels [121]. Such a system could be adapted to create, for instance, a stria vascularis-on-chip system. Introduction of endothelial cells and pericytes will alter cell–cell and cell–matrix interactions, and the additional supply of nutrients to areas deprived of physiological levels (due to the growth and large size of organoids), might beneficially affect maturation in an inner ear organoid system.

Most recently, it has been shown that forms of genetic hearing loss are linked to autoinflammation in the inner ear [122]. Like many other organoid models, the utility of in vitro inner ear organoid systems may be expanded by incorporating key immune cell populations, perhaps generated from autologous iPSCs. A prime example of immune cell incorporation into organoid models has been the use of microglia in cerebral organoids [123, 124]. Microglia are the resident immune cells of the brain, and recently, researchers have successfully derived microglial cells by guiding hPSCs into the mesodermal lineage. In parallel, brain organoids were induced and seeded with microglia using a simple co-culture approach. The timing of seeding was chosen to reflect the timing of microglial migration to the brain during normal development. Similarly, a parallel-induction and seeding approach of immune cells could be employed to incorporate macrophages into inner ear organoids and set the stage for inflammation studies [125].

## Conclusions and future directions

**Fig. 2:**
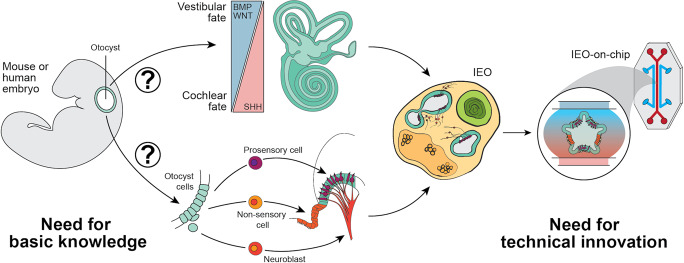
A roadmap to improve current inner ear organoid models. Firstly, there is a great need for basic knowledge on the patterning of the otocyst to further control inner ear organoid (IEO) development. The signaling molecules and pathways involved in the patterning processes of vestibular versus cochlear (top), and cell type fate determination (bottom), should be unraveled. These processes should be mimicked in inner ear differentiation protocols to reach a functional unit of cell types within the IEO. Secondly, to bridge the gap to translational research, a more controlled environment with high-throughput possibilities could be achieved by technical innovations, like the incorporation of microfluidics in the system, resulting in an inner ear organoid-on-chip.

As we have discussed, major breakthroughs have been made in inner ear modeling using hPSCs, yet current models are limited in their applications for translational research. To induce greater cell diversity in inner ear organoid models, it is essential to identify key regulatory pathways that play a role in determination of vestibular versus cochlear, epithelial versus mesenchymal, and sensory versus non-sensory cell fate decisions. Manipulation of these pathways should be systematically incorporated into current differentiation protocols (Fig. 2). More fundamentally, cell type specific markers that can distinguish between vestibular versus cochlear cell populations at every stage of development are lacking. In this regard, single-cell resolution atlases of the developing inner ear will be an important tool to advance inner ear organoid platforms. Initial single-cell mapping efforts have focused on maturation of the cochlea, specifically, the organ of Corti epithelium [73, 126–129]. To improve PSC-based otic induction strategies, however, it will be beneficial to have broader single-cell reference atlases for the inner ear at various developmental timepoints. For instance, researchers should perform single-cell analysis on earlier stages of development (i.e., E8.5–E11.5) to encompass the critical period of otocyst axial and sensory versus non-sensory patterning. In addition, single-cell atlases including both cochlear and vestibular compartments of the inner ear would allow for comparative analysis between the two organ systems. Recent work on foregut differentiation has demonstrated how single-cell RNA-sequencing data from critical periods of development can be used to construct more refined cell lineages and infer epithelial–mesenchymal cell interactions [113]. A similar approach could be applied to better define otic induction in the mouse embryo. Moreover, single-cell genomics data from human fetuses—as have been generated recently for the human fetal liver, skin, and inner ear [73, 130]––could be used to refine human otic induction methods. Together with the introduction of technical innovations, such as co-cultures and microfluidics, a more complete, scalable, and reproducible inner ear organoid model could be used to study inner ear dysfunction and sensory recovery for tomorrow’s medicine.
